# Wild Herbivore Grazing Enhances Insect Diversity over Livestock Grazing in an African Grassland System

**DOI:** 10.1371/journal.pone.0164198

**Published:** 2016-10-26

**Authors:** James S. Pryke, Francois Roets, Michael J. Samways

**Affiliations:** Department of Conservation Ecology and Entomology, Stellenbosch University, Private Bag X1, Matieland, 7602, South Africa; University of Pretoria, SOUTH AFRICA

## Abstract

Southern Africa’s grassland biodiversity is threatened by habitat transformation such as commercial forestry. Ecological networks (ENs) have been instigated to alleviate the pressure of habitat transformation on local biodiversity. ENs are large scale webs of corridors and patches of natural vegetation criss-crossing production landscapes that can simulate conditions in protected areas (PAs). Many ENs have lost many native large mammal species, which have been replaced by domestic livestock to retain natural grazing dynamics, which could have an impact on the long-term value of ENs for insects. Here we compared dung beetle, butterfly and grasshopper diversity in ENs across a landscape mosaic of timber plantations, where 1) wild megaherbivores were maintained, 2) in ENs where these herbivores were replaced by livestock and, 3) in a nearby World Heritage PA which retained its natural complement of megaherbivores. Sites in the PA far from any plantation were similar in composition to those in the wild grazed EN. Presence of the wild grazers improved the alpha- and beta-diversity of all focal insect taxa when compared to domestic grazing. Furthermore, species composition shows significant differences between the two grazing systems indicating that an assemblage of native large mammals facilitates insect diversity conservation. We support the maintenance or introduction of large native mammals in ENs or similar conservation areas in production landscapes to simulate the ecological conditions and natural heterogeneity in nearby PAs.

## Introduction

Globally, solutions are needed to alleviate the biodiversity crisis without compromising agriculture or timber production [1]. Africa’s iconic megafauna, like other terrestrial mammals, are in decline due to habitat transformation and fragmentation. This could lead to cascading effects on ecosystem function and declines in species that interact with these mammals [2]. Little is known about how biodiversity responds to different mammal species across a land use intensity gradient [3]. This leads to the debate on whether land sharing (landscape softening schemes that allow wildlife to interact, use and move through production areas) or land sparing (land set aside in production areas for biodiversity conservation) [4] is best. In large production systems land sparing seems best, as it promotes the persistence of sensitive species [5], although both schemes are likely to contribute significantly towards biodiversity conservation [6]. One system of land sparing is the creation of ecological networks (ENs), a web of landscape-scale connected corridors and nodes across transformed landscapes [7].

ENs effectively conserve many insect and plant species [8,9]. In the design of ENs, edge effects need to be taken into consideration as the effectiveness of corridors is determined by the size of the edge effect [10]. This means that for ENs to be effective in conserving invertebrates, corridors need to be wide enough to provide natural habitat not affected by edges [11]. Recent work has shown that natural spatial heterogeneity [12], control of alien plants [13], and appropriate fire and grazing management that mimic natural cycles [14] are also vital considerations when planning ENs.

Here, we study a system that is naturally characterised by a mix of woody and grassland areas, much of which has been converted to *Eucalyptus* plantations [10]. To alleviate the pressures on the local biodiversity, ENs were established in one area (Dukuduku and Nylasi) where the native large mammal assemblage is able to freely roam between the ENs and the neighbouring protected area (iSimangaliso Wetland Park, a World Heritage Site). Nearby, in another plantation (Kwambonambi), ENs have also been established, but because of the loss of large indigenous grazers, livestock grazing has been introduced in ENs to retain natural grassland dynamics [10]. If grasslands remain un-grazed, competitive woody species begin to dominate, often reducing the overall number of species in system, and when left for a long time, particularly in the absence of fire and during periods of high temperatures, grassland begins succession towards woody thicket [15,16]. Although there are concerns that domestic livestock grazing is worse for the environment than native megaherbivore grazing [17,18], at moderate grazing levels it is still more beneficial than no grazing [19,20]. The use of livestock grazers within conservation is a complex issue, as livestock often bring in diseases that affect native wildlife [21], are present at different densities to native grazers [17], and also compete with native grazers where these are still present [22]. Domestic grazers are not as mobile as wild grazers, with domestic animals congregating in certain areas, particularly waterways and pools [23]. Furthermore, areas under heavy use are trampled, leading to soil erosion and other disturbances [15].

It is well known that grazing is important for the maintenance of grassland insect diversity [24,25, 26]. From various grazed systems around the world, overgrazing has been shown to negatively affect vegetation structure [27], reduce the presence of food plants or pollination sources [28], and lead to invasion by exotic plants [29]. Within the South African grassland context, grasshoppers are more responsive to management (fire, grazing and mowing) than to landscape or patch variables (i.e. patch size, context, etc.) and so need grazing to maintain their diversity and function within grasslands [30,31]. Yet, grasshopper diversity seems to show little change when livestock grazing is compared to that of wild ungulates [32,33], with the disturbances caused by livestock grazing seemingly mimicking those of wild ungulate grazing. Butterflies on the other hand are more sensitive to the presence of grazers in general [3]. Well managed livestock grazing in a Kenyan savanna mimicked that of nearby wild ungulate grazing [34], indicating that for butterflies, grazing intensity of livestock is more important than the type of grazer.

Dung beetles have a more direct link to large mammals that co-inhabit the grasslands or savannas, as they are reliant on the dung produced, and the loss of large vertebrates is a major concern for dung beetle conservation [35]. An effective dung beetle conservation management strategy when wild large mammal grazers are lost to systems in Italy is to allow livestock to graze the land at light to moderate levels [36]. When dung beetle diversity in native wild mammal grazed grasslands is compared with exotic livestock grazed grasslands in South Africa and the Mediterranean, there is either little difference in species richness or higher species richness where wild animals graze, but assemblage composition is often dissimilar [37,38]. This is likely due to generalist species being conserved in these livestock systems, yet dung specialist species (due to niche segregation among dung beetles) are lost [39,40].

Use of domestic livestock as a replacement for wild herbivores [37] and the construction of ENs [11] both have been shown to be important for insect conservation in African grasslands. Yet to improve the conservation significance of ENs, we need to know how best to manage them and where conservation efforts should be concentrated. To address this question, we determine here how dung beetle, butterfly and grasshopper species richness, beta-diversity and species composition are most influenced by certain landscape variables, especially whether sites are in ENs or PAs, the effect of corridor size, and the distance to timber plantations, and also the importance of wild megaherbivores in the ENs. The loss of land to timber plantations is expected to have a major influence on arthropod diversity along with the replacement of the dominant large mammalian grazers by domestic livestock. Native mammal grazers may also play a more complex role in the function and maintenance of these grasslands than previously thought, and we also investigate the extent to which this is the case.

## Methods

### Study area and design

**Fig 1:**
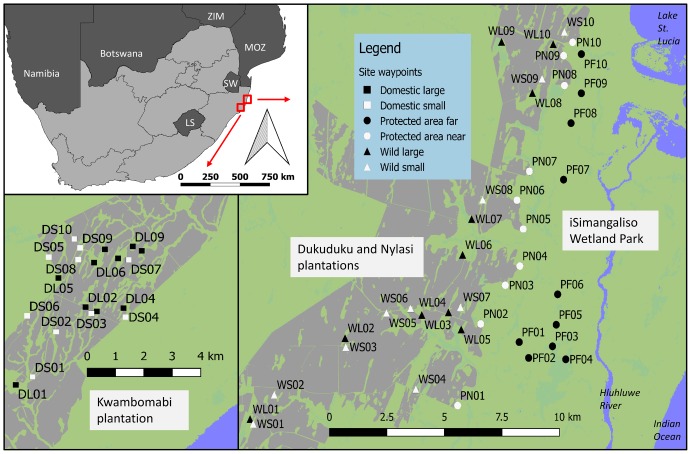
Map showing the location of the Dukuduku and Nyalazi plantations which have wild animals roaming through them, as well as the protected area (PA) of iSimangaliso Wetland National Park, and Kwambonambi plantation which is unconnected to a PA and has domestic livestock grazing in the ecological network (EN)). DL = domestic livestock sites in large corridors, DS = domestic livestock sites in small corridors, WL = native megaherbivore sites in large corridors, WS = native megaherbivores sites in small corridors in ENs, PN = site in PA near plantation, PF = sites in PA far from plantation, Plantation blocks are shown in dark grey, open areas in green, waterbodies in blue and communal areas in red, LS = Lesotho, SW = Swaziland and MOZ = Mozambique. Map generated from the South African Government, Department of Environmental Affair’s land cover map (available from: www.environment.gov.za/mapsgraphics).

This study took place within the Indian Ocean coastal belt biome, located in the northern coastal areas of Zululand, KwaZulu-Natal, South Africa, with only 25% of this vegetation type under formal protection [41]. This area is topographically flat, has very sandy soils, and is dominated by extensive commercial forestry plantations (mostly *Eucalyptus* spp.), but through Forestry Stewardship Council (FSC) regulations, large tracts of land within these plantations remain unplanted in the form of large, remnant patches and corridors creating ecological networks (ENs). Two plantations in the region, Nyalazi (28°39 S; 32°60 E) and Dukuduku (28°59 S; 32°42 E) border the iSimangaliso Wetland Park (a World Heritage Site) (28°32 S; 32°39 E) (Fig 1). These plantations are unique in that they have no fence line on the border with this large park, and many large animals, including the African elephant *Loxodonta africana*, white rhino *Ceratotherium simum*, African buffalo *Syncerus caffer*, plains zebra *Equus burchellii* and blue wildebeest *Connochaetes taurinus* move freely through and use the ENs within these plantations. Permission was granted by iSimangaliso Wetland Authority, Ezemvelo KZN Wildlife, SiyaQhubeka and Mondi South Africa to sample on their holdings.

Insect sampling took place during January and February 2013 on Nyalazi and Dukuduku, where wild native herbivores are the dominant grazers (wild grazed EN), as well as on Kwambonambi (28°66 S; 32°16 E), a nearby plantation 22 km south of Dukuduku where domestic cattle have replaced the wild grazers within the plantation (domestic grazed EN). Further sampling was conducted on the western shores of the iSimangaliso Wetland Park (wild grazed PA) (Fig 1). In total, 60 sampling sites were established, with 20 sites in each of the wild and domestically grazed EN sites, and 20 sites in the PA. The twenty sites per EN type were further split into 10 sites in large corridors (200–1300 m wide) and 10 sites in small corridors (30–150 m wide). The 20 sites that were established in the PA were split into 10 sites near the plantation edge (30–150 m) and 10 sites far from the plantation edge (1–3.8 km). Sites were chosen to be as widespread as possible, as well as accessible. At the centre of all sites, pitfall traps were dug in and baited to sample dung beetles, from this point 2x 200 m transects were established running in opposite directions for butterfly and grasshopper sampling. Plots were established in the middle of corridors and all transects avoided going closer than 30 m to plantation edges [11]. Sampling was conducted in an intensive sampling effort in January and February 2013 during which all sites were sampled once. At each site, the type of grazing, whether the site was located in an EN or PA, distance to nearest plantation, and corridor width were recorded. For the PA sites, corridor width was measured as the shortest distance between the plantation edge and the nearest natural barrier (in this case the Hluhluwe river or Lake St Lucia; ranging from 2.6–6.1 km wide) (Fig 1).

Cattle grazing intensity within the domestic EN system is difficult to quantify as this is an open communal grazing system with several different human communities using the land. As such, there is no quantifiable way of knowing how many heads of cattle are on the land. Furthermore, these areas are grazed differentially with over-grazing near communal access points, while interior sites are often under-grazed. Within the wild herbivore grazed areas, it is equally difficult to quantify grazer densities due to the park being about 3 300 km^2^ and the wild game species are able to roam freely across the whole PA. The grass species composition and height are similar between the wild grazed EN and the PA [9], suggesting that grazer densities are roughly equal. A management report on the graze quality of the two ENs systems suggests that the domestically grazed EN is about 25% under-grazed, 50% slightly over-grazed and 25% well managed, while the wild EN is 75% under-grazed and 25% well-managed [42]. This suggests that there is a slightly higher grazing intensity in the domestically grazed ENs compared to the other sites here.

## Arthropod Sampling

At each site, dung beetles were sampled using four baited pitfall traps, spread >5 m from each other forming a square. Each pitfall trap consisted of a 2.5 L plastic bucket that was buried so that its rim was flush with the ground and had 500 ml of 1:1 ethylene glycol mix. Each trap had a wire harp above it, from which hung a cloth bag containing ca. 100 ml of dung [43]. At each site, four different dung types were used: pig, cow, wildebeest and elephant. Pig dung captures most species of dung beetles present [43], while cow and wildebeest represented the most common domestic and wild ruminant grazers respectively. Elephant dung is known to attract specialist species and so was also included [35]. Each bait bag was made using the same batch of dung collected in a single day. All bait bags were frozen and only defrosted on the day of use. This allowed us to standardise dung volatiles. Traps were left open for 2 days, after which the dung bait dried out and the volatiles become inactive. Dung beetle specimens were collected, preserved and identified to genus level [44], and assigned to morphospecies.

Butterflies were sampled using the Pollard walk method [45], where two observers walked in the opposite direction from each other, but never more than 50 m from the centre of the site, recording all the butterfly individuals encountered. Care was taken to record abundance without recounting the same individuals. Once an individual left the observer’s visual range, it was assumed it would not come back, and so any new individuals that entered the visual range of the observers were counted as new individuals. Each observation took an hour, and was only done on sunny, windless days between 08h30 and 15h30. A reference collection was made to assist with field identification, with most species identified on the wing. Some were captured with an aerial hand net and later identified to species level [46,47]. Butterflies were also classed into two groups, one nested inside the other: those species associated with grasslands (i.e. those recorded in the literature as having only been found in grasslands [46,47]), and all butterflies encountered.

Grasshoppers were sampled at each site by making 400 sweeps using a 40-cm sweep net. One sweep was a single back and forth movement through the grass. All flushed grasshoppers (i.e. those that flew away ahead of the sweep net) were pursued and captured. This method is effective for sampling most grasshoppers at moderate to high densities in short grass [48]. Grasshopper samples were frozen and adults were identified to the lowest possible taxonomic level [49]. Voucher specimens are in the Entomology Museum, Department of Conservation Ecology and Entomology, Stellenbosch University.

### Statistical analyses

Generalized Linear Mixed Models (GLMMs) were calculated using the MASS package in R [50,51] using the penalized quasi-likelihood estimation method and data fitted to a Poisson distribution [52]. These data were tested for spatial autocorrelation using a semivariogram. When a random, dummy variable was exponentially correlated to longitude and latitude it improved the semivariogram [53]. Correlated longitudinal and latitudinal data were nested within the three distinct areas (iSimangaliso, Kwambonambi as well as Dukuduku and Nylasi combined) and this was used as the random variable to overcome spatial autocorrelation in the data. These analyses were done for species richness of all collected insects, and for the four taxa separately (dung beetles, butterflies, grassland-associated butterflies and grasshoppers). Models were constructed with grazing type, whether sites were in an EN or PA, corridor width and distance to plantations as fixed variables, and exponentially correlated longitude and latitude nested in the sampling area as the random effect. Forward selection was then used to determine the interaction between these factors. We were unable to calculate the interaction between grazing type and EN vs. PA due to the missing combination variable (i.e. domestically grazed PA, which does not exist by definition). Alpha diversity (rarefied observed species richness) was calculated using the BAT package in R [54]. Alpha-diversity was calculated based on the observed data rarefied using 50 rarefication runs for all analyses except the grasshoppers, which due to low sampled numbers were only rarefied 10 times.

To test the similarities in arthropod assemblage composition, Permutational multivariate analysis of variance (PERMANOVA) in PRIMER 6 [55] was done, with effect of grazing type, whether sites were in a EN or PA, corridor width, and distance to plantations as fixed variables, and elevation as a random variable. A second full model was constructed with the first-order interactions included in the models. These models were constructed for all focal taxa and each taxon separately. Assemblage composition F- and p- values were calculated using 9999 permutations [56]. The weight of common species was reduced using square-root transformation on the data, and analyses were performed using Bray-Curtis similarity measures [57]. Differences were also explored using canonical analysis of principal coordinates (CAP) in PRIMER [58].

Beta-diversity refers to species change between sites, yet two processes shape these changes, either species replacement or species loss [54]. To understand which of these two components of beta-diversity are driving the between-site variation seen in the PERMANOVA, we calculated beta-diversity and partitioned beta-diversity (changes due to species replacement and species loss separately) for each of the six site types for all taxa and per taxon in the BAT package [54]. This procedure calculates three forms of beta-diversity, namely: β_total_ = total beta-diversity (both replacement and richness components combined), β_repl_ = replacement component of this diversity (i.e. beta-diversity due to species turnover) and β_rich_ = the richness component (beta-diversity due to species loss or gain) [54]. Higher β_repl_ suggests natural turnover is responsible for observed beta-diversity changes, while higher β_rich_ suggests that differences in species richness are more responsible for beta-diversity changes (normally due to ecological disruption at one of the sites). We used the Sørensen beta-diversity measure, which is based on abundance data, and each of the measures is based on all sites combined per treatment.

## Results

In total, 27 121 individual adult insects were sampled here from 152 observed species. We sampled 23 219 dung beetle individuals from 63 species, 3 628 butterflies from 55 species and 5 families, 2 828 grassland associated butterflies from 21 species and 4 families, and 279 grasshoppers from 34 species and 6 families. Species accumulation curves reached an apparent asymptote for all groups, except grasshoppers which reached a near asymptote (S1 Fig).

**Fig 2:**
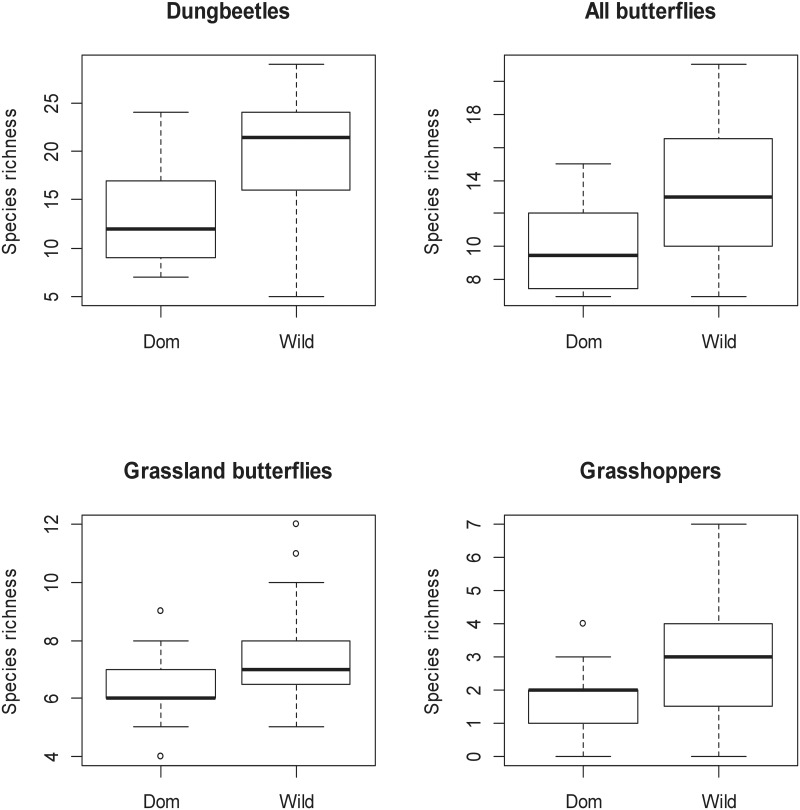
Boxplots of the species richness of each focal taxon for sites under domestic grazing (Dom) and wild herbivore grazing (Wild). All graphs show significant results (Table 1).

**Fig 3:**
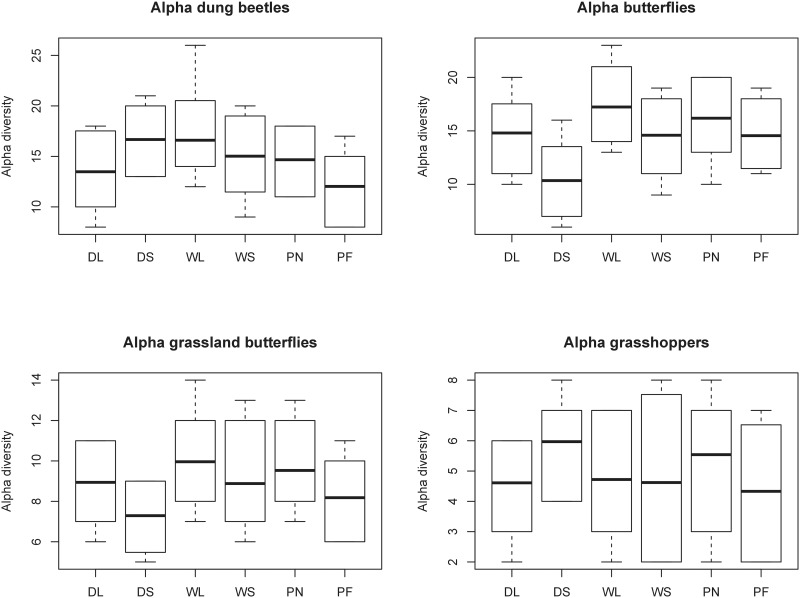
Results of rarefication for the alpha-diversity of (A) dung beetles (738 individuals and 100 runs), (B) all butterflies (521 individuals and 100 runs), (C) grassland butterflies (365 individuals and 100 runs) and (D) grasshoppers (25 individuals and 100 runs). D = domestic livestock in ecological networks (EN), W = native megaherbivores in ENs, L = large corridor, S = small corridor, P = protected area, N = near plantation, F = far from plantation.

Areas with wild grazers always had significantly higher insect species richness compared to areas under domestic grazing for all taxa combined and for each focal taxon separately (Table 1; Fig 2). Overall, the PA had significantly higher species richness than ENs, although when analysed per taxon, this result was only seen for butterflies overall (Table 1). Corridor width was positively correlated to overall species richness, but this effect was not seen for any of the individual groups (Table 1). There were only two significant interactions and they were both for grassland butterfly species richness. These interactions were between grazing type and distance to plantation, as well as grazing type and whether the sites were in an EN or PA (Table 1). Rarefied alpha-diversity for dung beetles was similar across the site types, although the sites in the PA far from the plantation were the lowest (Fig 3). Butterflies overall showed lowest alpha-diversity in small domestically grazed sites, and highest alpha-diversity in the large wild grazed sites. However, when forest species where removed, the wild small corridors also had high alpha-diversity (Fig 3). Grasshoppers showed little change in alpha-diversity across all sites (Fig 3).

**Table 1:** Results of main effect tests on species richness and species composition for grazing type (whether domestic or wild herbivores are present), location of sites in an ecological network or protected area, width of corridor, distance to plantation, and the interaction between these factors where possible. Species richness analyses used PQL generalized linear mixed models (with Poisson distribution and exponential correlation of geographical co-ordinates as a random effect) are shown here with χ^2^ values, while species compositional analyses used permutational multivariate analysis of variance (with elevation as a random effect) are shown with pseudo-F values.

Species richness	All groups	Dung beetles	All butterflies	Grass butterflies	Grasshoppers
Grazing type	5.31***	3.64***	3.17**	2.87**	2.64*
EN vs PA	2.30*	1.42	2.40*	0.36	0.21
Corridor width	2.01*	1.44	1.72	1.47	0.15
Dist to plantation	0.05	1.34	1.95	0.55	0.71
EN*CorrW	0.34	0.78	0.37	0.09	0.34
Graze*DistP	0.74	0.02	1.32	3.35**	0.13
Graze*CorrW	0.02	0.22	0.03	1.47	0.47
DistP*EN	0.85	0.48	1.17	2.28*	0.67
CorrW*DistP	0.63	0.32	0.90	0.61	0.07
**Species composition**					
Grazing type	3.18**	2.94**	2.44**	3.17**	5.20***
EN vs PA	1.29	2.18*	1.93*	1.71	0.72
Corridor width	1.17	1.28	1.40	1.25	1.47**
Dist to plantation	1.10	1.31	0.71	0.91	1.63
Graze*CorrW	0.89	0.80	0.98	0.74	0.65
Graze*DistP	1.02	1.09	0.69	0.69	0.48
EN*DistP	1.49	1.63	0.83	0.38	0.97
CorrW*DistP	1.02	1.10	0.68	0.31	0.48

Grass butterflies = grassland butterflies, Graze = Grazing type, CorrW = Corridor width, DistP = Distance to plantation, EN = Ecological network, PA = Protected area.

* p <0.05,

** p <0.01,

*** p <0.001.

**Fig 4:**
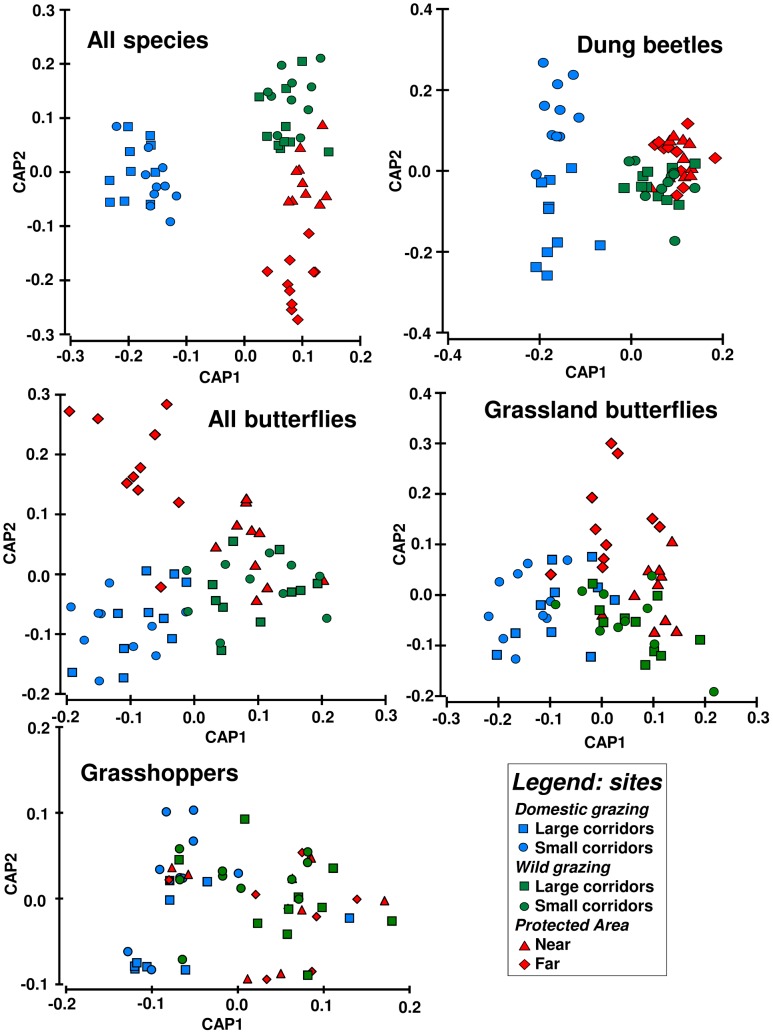
Canonical analysis of principal coordinates ordination (CAP) for all the sites, sites in the protected area (red), wild EN (green) and domestic EN (blue); as well as those from small corridors (circles), large corridors (squares), near the plantations (triangle) and far from the plantations (diamond).

Type of grazing was a significant driver of species assemblage composition for all groups together, and each group separately (Table 1; Fig 4). There were also influences between the location of sites in ENs vs. PA for the dung beetle and overall butterfly assemblages (Table 1), although these seem related to the difference in grazing regimes between the two different kinds of ENs (Fig 4). Corridor width also significantly influenced the grasshopper assemblage (Table 1; Fig 4). There were no significant interactions in the similarity of species assemblage composition for any group (Table 1).

Beta-diversity of dung beetles was higher between domestically grazed and wild grazed sites (average β_total_ = 0.732) (S1 Table). This was mainly due to a higher β_rich_ component (average β_repl_ = 0.129; average; β_rich_ = 0.603). The PA sites far from the plantation also had a higher β_total_ compared to other wild grazed sites (average β_total_ = 0.434), also due to a higher β_rich_ component (average β_rich_ = 0.312). Butterflies (both the whole assemblage and the grassland species) differed little between site types in their β_total_ (average β_total_ for all butterflies = 0.374; β_total_ for grassland butterflies = 0.326). The small difference that was present was driven by the β_repl_ (average β_repl_ for all butterflies = 0.276; average β_repl_ for grassland butterflies = 0.216) rather than β_rich_ (average β_rich_ for all butterflies = 0.098; average β_rich_ for grassland butterflies = 0.110). Grasshoppers showed higher beta-diversity between domestically grazed and wild grazed sites (average β_total_ = 0.581). This was equally driven by both β_repl_ (average β_repl_ = 0.368) and β_rich_ (average β_rich_ = 0.212) components (S1 Table).

## Discussion

### Impacts of grazing on insect diversity

Our results show that having wild grazers within the ENs enhances the insect biodiversity value, with a consistent result for dung beetles, butterflies and grasshoppers for measures of species richness, beta-diversity and assemblage composition. With the study design here, it is difficult to separate the grazing regime from the area sampled. This is particularly the case for the domestic- vs. wild-grazed comparisons. Nevertheless, it seems that the presence of wild grazers in general does promote historic insect diversity [59]. This result emphasises that a variety of megaherbivores creates many niches for insects, highlighting that these interactions are a fundamental part of this system and are critical to conserve [3,16,26,35,60].

Dung beetle diversity was driven by type of grazing regime. This adds to the concerns that some dung beetle diversity will be lost when native large mammals are no longer present in the system [35]. The beta-diversity of dung beetles in this area was high, with greatest rates of species turnover between the domestic and wild grazed areas. This was most likely driven by the loss of specialist dung beetle species and not replacement. As dung beetles and the native large grazers have coevolved in these systems, it is not surprising that specialist species are responding to only a few wild mammal species [39]. Butterflies also showed a strong association with the presence of wild grazers. This is most likely due to the persistence of more plant species which serve as food for adults (pollen and nectar) and larvae (food plants), a result of natural associations and better ecosystem function under wild native mammal grazing [28]. The whole butterfly assemblage, and not just the grassland species, showed moderate species turnover, driven by a high rate of species replacement. This indicates that butterflies are well conserved in both types of ENs sampled here. The response of grasshoppers to the presence of wild grazers here is more surprising, as other studies in Europe and Africa have shown that they are not much influenced by the type of grazer [32,33]. This is most likely due to a greater variety in the types of grazing in the wild grazed area, creating more niche opportunities, or this might be due to differential grazer intensities between the two ENs [17]. More likely, these differences and conclusions are related to grazing intensity, which greatly affects vegetation structure and composition, and so determining local grasshopper assemblages whether the grazing is done by native or domestic megaherbivores [32,61].

The role of domestic grazing in the conservation of grassland insects remains unclear. Even when done only by domestic animals, grazing is still important for the maintenance of grasslands [19,20], and thus important for most arthropod species, with focus also being given to grazing intensity [32,34,61], with occasional exceptions [62]. The concern is that some of the specialist insect species have been lost from these systems due to the lack of habitats created by wild grazers. What is unclear from these results is whether it is the absence of specific mammalian species from the domestic grazing system or a more simplified grazing community that is causing this change. For example, zebra are long grass feeders and wildebeest are short grass feeders, and the two interact to create grazing lawns, which many other species also use [63]. This raises the possibility that mixed grazing in domestically grazed areas (i.e. cattle, sheep and goats in one open system) might be better than a single grazer type (just cattle in our system), although this would still need to be verified.

### Relative value of context and distance of plantation on insect diversity

Sites in the PA far from any plantation were generally similar in species richness, alpha-diversity, beta-diversity and assemblage composition to those in and around the ENs. There were some differences between the ENs and PA for the dung beetle and butterfly assemblage compositions, but these seem to arise from differences in the grazing systems and these wild grazed sites grouped together in CAP analyses. Dung beetles showed a high variation in alpha-diversity in deep PA sites and a high β_rich_ between deep PA sites and the other wild grazed sites. This suggests that some species have been lost between these sites. This is likely due to the movement patterns of mammals. For example, elephants use shelter provided by the exotic trees and thickets in and around the ENs in preference to overall lower number of shelter patches in the PA [64]. We have also observed this general trend for other large animals in the area, although this was not empirically tested. Therefore, the dung beetles might not be detected in the PA as much, simply because the large mammals are scarcer in the far PA sites.

Distance to plantation and corridor width had very little effect on arthropod diversity. This is likely due to the large size of the corridors in the ENs studied here, which are known to be rich in native biodiversity [65]. This emphasizes the point that well-designed ENs are able to conserve arthropod diversity. Grasshoppers in particular show large changes in assemblage composition in large compared to small corridors, with small corridors characterized by early successional habitat suitable for certain specialist species [30]. Here, butterflies showed a high β_repl_ value across the whole study area. This is expected as this region is a butterfly hotspot [66], and β_repl_ is essentially a function of gamma diversity [67].

### Conclusions and management implications

Overall our results show that for insect conservation, it is not just spatial attributes that maintain high insect diversity (here in the form of the ENs). Management strategies are also important, especially the retention of wild mammals. We support the maintenance or reintroduction of native large mammal herbivores into ENs and other conservation areas where possible. Furthermore, having a highly complex native herbivore assemblage is important for creating as many different niches as possible for as much of the insect assemblage as possible [59,60,61]. This allows ENs to mimic the PAs better than those under domestic grazing. Where this is not possible, grazing by domestic livestock, and in particular by a range of different species with different grazing habits, is still encouraged as this maintains the grassland and conserves a greater diversity of grassland insects and other invertebrates [65].

## Supporting Information

S1 Fig Rarefication curves. Rarefied species accumulation curves for dung beetles, all butterflies, grassland butterflies and grasshoppers. Curves represent observed species (black circles), Chao2 (grey triangles) and jacknife2 (open squares).
(PDF)

S1 Table Beta-diversity similarity between sites. Beta-diversity (using Sørensen index) between the six different sampling locations for various taxa (β_total_), with distances partitioned into beta-diversity due to richness (β_rich_) and replacement (β_repl_).
(PDF)
