# Supplementary material for: Wild Herbivore Grazing Enhances Insect Diversity over Livestock Grazing in an African Grassland System
Source: PLoS One. 2016 Oct 26;11(10):e0164198. doi: 10.1371/journal.pone.0164198 (PMC5082622; doi:10.1371/journal.pone.0164198)
Supplement: S1 Fig — Rarefied species accumulation curves for dung beetles, all butterflies, grassland butterflies and grasshoppers. Curves represent observed species (black circles), Chao2 (grey triangles) and jacknife2 (open squares). (PDF) [file pone.0164198.s001.pdf]

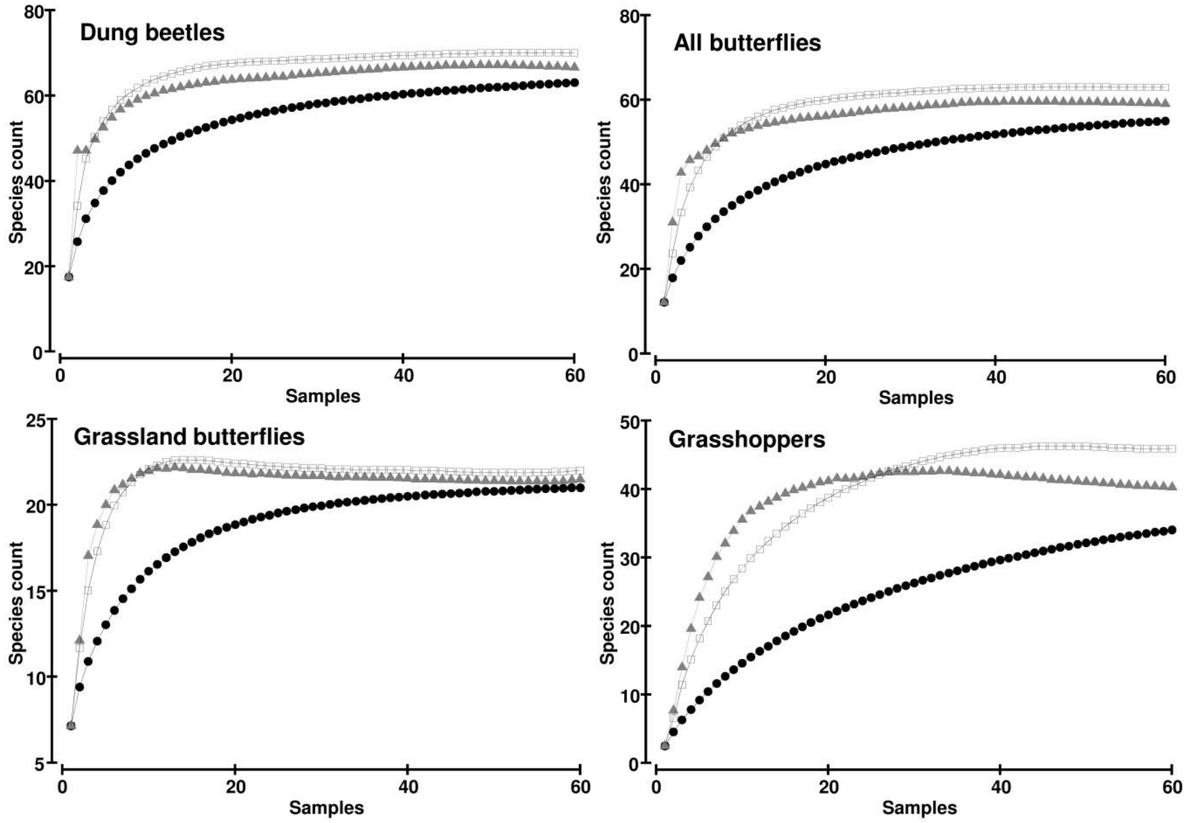

S1 Fig. Rarefied species accumulation curves for dung beetles, all butterflies, grassland butterflies and grasshoppers. Curves represent observed species (black circles), Chao2 (grey triangles) and Jacknife2 (open squares).
