# Supplementary material for: Wild Herbivore Grazing Enhances Insect Diversity over Livestock Grazing in an African Grassland System
Source: PLoS One. 2016 Oct 26;11(10):e0164198. doi: 10.1371/journal.pone.0164198 (PMC5082622; doi:10.1371/journal.pone.0164198)
Supplement: S1 Table — Beta-diversity (using Sørensen index) between the six different sampling locations for various taxa (βtotal), with distances partitioned into beta-diversity due to richness (βrich) and replacement (βrepl). (PDF) [file pone.0164198.s002.pdf]

S1 Table. Beta-diversity (using Sørensen index) between the six different sampling locations for various taxa (β_{total}), with distances partitioned into beta-diversity due to richness (β_{rich}) and replacement (β_{repl}).

		Dung beetles (above diagonal) / Grasshoppers (below diagonal)					
		**DL**	**DS**	**WL**	**WS**	**PN**	**PF**
β_{total}	**DL**		0.541	0.725	0.781	0.753	0.859
	**DS**	0.754		0.609	0.638	0.677	0.811
	**WL**	0.711	0.689		0.231	0.208	0.483
	**WS**	0.727	0.778	0.339		0.308	0.450
	**PN**	0.722	0.750	0.446	0.359		0.369
	**PF**	0.605	0.747	0.328	0.309	0.446	
β_{repl}	**DL**		0.134	0.039	0.078	0.036	0.027
	**DS**	0.632		0.221	0.223	0.238	0.168
	**WL**	0.371	0.244		0.198	0.147	0.143
	**WS**	0.455	0.395	0.264		0.279	0.139
	**PN**	0.532	0.444	0.286	0.272		0.085
	**PF**	0.349	0.380	0.235	0.291	0.376	
β_{rich}	**DL**		0.407	0.686	0.703	0.717	0.832
	**DS**	0.123		0.388	0.415	0.438	0.643
	**WL**	0.340	0.444		0.032	0.061	0.340
	**WS**	0.273	0.383	0.074		0.029	0.311
	**PN**	0.190	0.306	0.161	0.087		0.284
	**PF**	0.256	0.367	0.092	0.018	0.069	

		All butterflies (above diagonal) / Grassland butterflies[1] (below diagonal)					
		**DL**	**DS**	**WL**	**WS**	**PN**	**PF**
β_{total}	**DL**		0.281	0.286	0.263	0.435	0.422
	**DS**	0.247		0.434	0.351	0.541	0.458
	**WL**	0.250	0.390		0.211	0.365	0.466
	**WS**	0.181	0.296	0.183		0.316	0.407
	**PN**	0.362	0.465	0.335	0.264		0.380
	**PF**	0.352	0.400	0.469	0.369	0.324	
β_{repl}	**DL**		0.173	0.255	0.169	0.241	0.400
	**DS**	0.081		0.356	0.336	0.454	0.328
	**WL**	0.230	0.205		0.147	0.201	0.414
	**WS**	0.110	0.200	0.092		0.216	0.291
	**PN**	0.269	0.391	0.223	0.242		0.165
	**PF**	0.269	0.155	0.406	0.216	0.149	
β_{rich}	**DL**		0.109	0.031	0.095	0.193	0.022
	**DS**	0.166		0.078	0.014	0.087	0.130
	**WL**	0.020	0.185		0.064	0.164	0.052
	**WS**	0.071	0.096	0.091		0.101	0.116
	**PN**	0.093	0.074	0.113	0.022		0.214
	**PF**	0.083	0.245	0.063	0.153	0.174	

D = domestic EN, W = wild EN, L = large corridor, S = small corridor, P = protected area, N = near plantation, F = far from plantation, [1]Forest associated species removed from these analyses.
